# Investigation of Serotype Prevalence of *Escherichia coli* Strains Isolated from Layer Poultry in Greece and Interactions with Other Infectious Agents

**DOI:** 10.3390/vetsci9040152

**Published:** 2022-03-23

**Authors:** Dimitrios Koutsianos, Labrini V. Athanasiou, Dimitris Mossialos, Giovanni Franzo, Mattia Cecchinato, Konstantinos C. Koutoulis

**Affiliations:** 1 Department of Poultry Diseases, Faculty of Veterinary Science, School of Health Sciences, University of Thessaly, 43100 Karditsa, Greece; kkoutoulis@vet.uth.gr; 2 Department of Medicine, Faculty of Veterinary Science, School of Health Sciences, University of Thessaly, 43100 Karditsa, Greece; lathan@vet.uth.gr; 3 Laboratory of Microbial Biotechnology-Molecular Bacteriology-Virology, Department of Biochemistry and Biotechnology, School of Health Sciences, University of Thessaly, Biopolis, 41500 Larissa, Greece; mosial@uth.gr; 4 Department of Animal Medicine, Production and Health (MAPS), University of Padua, 35020 Legnaro, Italy; giovanni.franzo@unipd.it (G.F.); mattia.cecchinato@unipd.it (M.C.)

**Keywords:** commercial layers, *Escherichia coli*, layer breeder, predisposing factor, O-serogroup

## Abstract

Colibacillosis is the most common bacterial disease in poultry and it is caused by avian pathogenic *Escherichia coli* (APEC), which is assigned to various O-serogroups. Previous studies have shown that APEC strains are more often related to certain O-serogroups such asO78, O2 and O1. *E. coli* has been reported to act either as a primary or secondary agent in complicating other infections. The aim of this study was to investigate the occurrence of and characterize the O-serogroups of *E. coli* strains isolated from commercial layer and layer breeder flocks showing macroscopic lesions of colibacillosis and increased or normal mortality in Greece. Furthermore, we attempted to assess the interaction between infectious agents such as *Mycoplasma gallisepticum* (MG), *Mycoplasma synoviae* (MS), infectious bronchitis (IBV) and infectious laryngotracheitis (ILT) with *E. coli* infections in layer flocks with increased mortality. Our study revealed that in addition to the common serogroups (O78, O2), many other, and less common serogroups were identified, including O111. The O78, O111 and O2 serogroups were frequently detected in flocks with lesions of colibacillosis and increased mortality whereas O2, O88 and O8 were reported more commonly in birds with colibacillosis lesions but normal mortality rates. These data provide important information for colibacillosis monitoring and define preventative measures, especially by using effective vaccination programs because *E. coli* vaccines are reported to mainly offer homologous protection. Finally, concerning the association of the four tested infectious agents with *E. coli* mortality, our study did not reveal a statistically significant effect of the above infectious agents tested with *E. coli* infection mortality.

## 1. Introduction

*Escherichia coli* (*E. coli*) is a common member of the gut microbiome in birds. However, several strains—known as avian pathogenic *Escherichia coli* (APEC)—are implicated in avian colibacillosis, which has been described as the most common bacterial disease in poultry, having a serious economic impact on poultry production [1,2,3,4]. APEC is responsible for different systemic or localized infections [5] such as colisepticemia (characterized by the presence of fibrinous exudates in various organs) [6], respiratory infections and air sacculitis [7], swollen head syndrome [1], peritonitis/salpingitis/salpingoperitonitis [4,8], yolk sack infections in day-old chicks and skin infections (cellulitis) [6,9].

*E. coli* can be a primary pathogen, causing clinical disease; occasionally, other predisposing factors must be present to help *E. coli* express its pathogenic effect. Various infectious agents have been described to be complicated with *E. coli*. Many viruses, especially those with an effect on the respiratory system such as paramyxovirus (Newcastle disease) [10], coronavirus (infectious bronchitis) [11,12], metapneumovirus (avian rhinotracheitis or swollen head syndrome) [13,14], orthomyxovirus(influenza) [15,16] and laryngotracheitis virus(infectious laryngotracheitis) [17] can trigger avian colibacillosis. Viruses that can act in a similar way to immunosuppressive factors such as *herpesviruses* (Marek disease) or *circovirus* (chicken anemia virus) can stimulate colibacillosis just as many other viruses can also stimulate *E. coli* infections [1,18]. Bacteria such as *Mycoplasma gallisepticum* [1] or *Mycoplasma synoviae* [19], or parasites such as *Eimeria* or *Ascaridia* (or red mites) can also predispose to an *E. coli* infection [1,20].

The scope of this study was to investigate the serogroup prevalence of avian *E. coli* strains isolated from birds demonstrating colibacillosis lesions, characterizing the presence and frequency of different O-somatic antigens, in Greece. As other studies worldwide have already considered with the broiler production, our project was focused on flocks from layer production. Most of the studies related to broilers have revealed the prevalent role of O78, O2 and O1 serogroups as APEC strains [1,2,7,8]. Furthermore, a high diversity of O-serogroups have also been isolated from broilers suffering from colibacillosis and the presence of untypeable *E. coli* strains has also been established [1,2,7].

As it has been reported that the use of *E. coli* vaccines induces homologous immunity to the vaccine strain used, data on the *E. coli* serotype status of an area can support colibacillosis vaccination prevention schemes [21,22]. Furthermore, we evaluated the possibility that other infectious agents such as *Mycoplasma gallisepticum, Mycoplasma synoviae*, the infectious bronchitis virus and the infectious laryngotracheitis virus correlated with the presence of colibacillosis and increased mortality in hens [23].

## 2. Materials and Methods

### 2.1. Sampling

**Figure 1:**
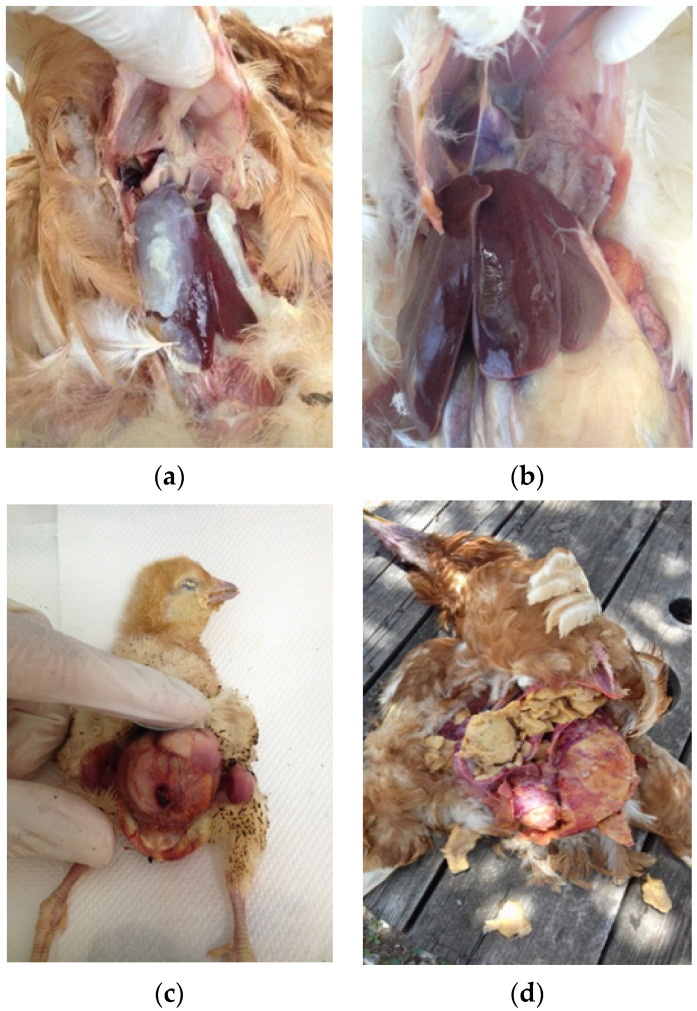
Macroscopic lesions indicating colibacillosis: (**a**) perihepatitis/pericarditis; (**b**) air sacculitis; (**c**) omphalitis; (**d**) peritonitis.

A total of 60 different farms and 140 flocks, including birds with colibacillosis lesions from diverse geographical areas in Greece were included in the present study. All samples were collected during the period 2016–2017. A mortality threshold of 0.1% per week was used to divide the flocks between ‘normal’ or ‘increased’ mortality (ISA management guide) [24]. A total of 231 *E. coli* isolates were recovered from organ samples, including the liver, pericardium, air sacks, yolk sacks and peritoneum/ovaries, which were collected from rearing pullets, commercial layers and layer breeders. During each farm visit, we collected 3–5 dead birds per flock according to farm availability. All birds were necropsied and in case there was a demonstration of colibacillosis macroscopic lesions such as perihepatitis, pericarditis, air sacculitis, omphalitis and peritonitis (Figure 1a–d), sampling was performed from the most apparent lesion.

### 2.2. Bacterial Isolation

Bacterial isolation was performed according to the following protocol. All samples were streaked on MacConkey agar (Bioprepare, Attica, Greece) and sheep blood agar (Bioprepare, Attica, Greece). Colonies that were phenotypically identified as *Escherichia coli* were further assessed for their biochemical properties by an analytical profile index (API) system. A total of 43 samples was evaluated by MICROGEN API GNA+B in a vet lab (Vet Analysis Lab, Athens, Greece) and, as a result, 24 samples were biochemically confirmed as *E. coli* and 19 needed further confirmation. Additionally, 207 samples, the 19 inconclusive and 188 new samples, were sent for an analysis to the Istituto Zooprofilattico Sperimentale della Lombardia e dell’Emilia Romagna ‘Bruno Ubertini’, Italy. Biochemical confirmation was conducted using the same protocol (MICROGEN API GNABatch 41362, expanuary 2018) and, for all inconclusive samples, a further biochemical confirmation was conducted using API 20E (BioMerieux, Batch 1004845000, exp. 10 February 2017). In total, 231 samples were confirmed as *E. coli*.

### 2.3. Serogroup Characterization

A total of 207 samples biochemically identified as *E. coli* in Italy were also serotyped by using a slow agglutination procedure in microtiter plates according to the Guinee agglutination method [24]. This technique is based on the agglutination that occurs when an *E. coli* culture is mixed with its homologous O antiserum [25,26].

The following agglutination protocol was used: a pure culture of *E. coli* isolates was obtained and it was used to prepare a bacterial suspension with a final concentration corresponding with a McFarland standard of 6.0. The suspension was treated at 100 °C for 1 h to obtain the final antigenic suspension. The heat treatment inactivated the K antigen. The bacterial suspension was then tested with a range of different antisera. Thirty different antisera that included the most common APEC serogroups (O1, O2, O5, O8, O9, O15, O18, O20, O22, O26, O45, O49, O55, O64, O78, O86, O88, O101, O103, O111, O113, O118, O128, O138, O139, O141, O147, O149, O153 and O157) were initially used for serotyping the *E. coli* strains. Each antiserum was diluted 1:40 and then inoculated in microtiter plates (U-bottom); a quantity of 100 µL of the antigenic suspension was then added in each well. The two suspensions were mixed and the plates were incubated at 37 °C for 18 h. After the incubation, the presence of agglutination was checked for positivity. Additionally, 24 samples that were confirmed in a laboratory (Vet Analysis Lab, Athens, Greece) as *E. coli* were sent for serotyping using agglutination testing to the Biovac laboratory (Biovac, Beaucouze, France).

### 2.4. Data Collection of Predisposing Factors

To assess the relation of other infectious agents with colibacillosis-increased mortality, the occurrence of other pathogens was investigated. Additionally, the following data were obtained: the results of serological testing through an ELISA, an applied vaccination program regarding these specific infectious agents and the presence of clinical symptoms indicating the disease. For the serological testing, 10 blood samples were collected from the flock at the same visit when the *E. coli* sampling took place. When possible, a second sampling after 3–4 weeks (pair samples) was performed. The flocks that were sampled for serological testing were randomly selected and organized according to the compliance of the farmer to allow for blood sampling. Blood samples from each flock were collected by awing branchial vein puncture. The samples were centrifuged at 4500 rpm for 10 min and the serum was collected.

### 2.5. Mycoplasma gallisepticum and Mycoplasma synoviae

None of the flocks sampled were vaccinated against either *Mycoplasma gallisepticum* or *Mycoplasma synoviae*. Therefore, the presence of positive antibody titers revealed an exposure to mycoplasma. The presence of 20% positive samples out of the 10 samples collected from each flock led to a characterization of an ‘infected’ flock (OIE terrestrial manual) [27]. The serum was tested by the ELISA method (Zoetis/Proflok MG kit, lot 1,501,808 and Zoetis/Proflok MS kit, lot 1500907). When the samples were negative, a second sampling was performed within 3–6 weeks to check for seroconversion. Therefore, we managed to characterize the infection status of 61 different flocks.

### 2.6. Infectious Bronchitis

Concerning infectious bronchitis, all sampled flocks were vaccinated against both mass and variant strains during the rearing period. To characterize the flock IBV infection status, we considered the flock antibody titers in combination with the flock vaccination program and the presence of clinical symptoms that indicated an IBV infection.

In order to assess the antibody titers against IBV with the ELISA method (Zoetis/Proflok IBV kit, lot 132880), we collected blood samples from 51 flocks. For 37 flocks, we managed to perform a paired sampling at an interval of 3–5 weeks whereas for 14 flocks, a single sampling procedure was performed. For flocks that were pair sampled, seroconversion was checked. In cases where a single sampling was performed, the mean titer was compared according to the Zoetis IBV guidelines for infection. Very high titers that were not in accordance with the vaccination program used were considered to be suggestive of an infection.

### 2.7. Infectious Laryngotracheitis

Regarding infectious laryngotracheitis (ILT) testing, both vaccinated and unvaccinated flocks were included in our study. In order to perform a characterization of the flock infection status for ILT, we used the serological profile of the flocks performed with the ELISA method (Zoetis/Proflok ILT kit, lot 123146) in combination with the flock vaccination program and the presence of clinical symptoms that indicated an ILV infection. The presence of antibody titers in cases where no vaccination was applied combined with the presence of clinical symptoms/lesions indicated an infection. When a vaccination had been applied, a rise in the mean antibody titer along with the clinical appearance were taken into consideration. The procedure for collecting the blood samples was the same as for IBV. Therefore, we collected blood samples from 51 flocks. For 37 flocks, we used pair sampling whereas for 14 flocks, a single sampling procedure was followed.

### 2.8. Statistics

The percentages of each serotype in birds with high and low mortality and among the types of birds as well as the percentages of the serogrouped samples from each geographic area tested were compared using Med Calc software. The association between specific pathogens and increased mortality due to an *E. coli* infection was tested using a chi-squared test. For all the analyses, the statistical significance was set at *p* < 0.05.

## 3. Results

### 3.1. O-Serogroup Characterization

In total, 231 samples were confirmed as *E. coli*. The total O-serogroup characterization associated with the flock mortality is shown in Table 1.

The majority of strains (71.4% in total) were untypeable by the tested range of monospecific antisera. In flocks with increased mortality, a lower percentage (69.3%) of untypeable strains was observed compared with 78.1% of flocks with a normal mortality.

For strains where typing was achieved, without taking into consideration the flock mortality trait, a total of 12 O-serogroups were found. The most prevalent serogroups were O78 (6.9%), O2 (6.5%) and O111 (6.5%); the remaining isolates were O88 (2.5%), O8 (1.7%), O45 (1.2%), O147 (0.8%), O103 (0.4%), O18 (0.4%), O15 (0.4%), O5 (0.4%) and O19 (0.4%).

With reference to the division of samples according to the flock mortality trait, 9 serogroups were related to the increased mortality and 7 to the normal mortality group. The most prevalent serogroups between the isolates were: (a) O78 (9.1%), O111 (8.5%) and O2 (6.2%); and (b) O2 (7.2%), O88 (3.6%) and O147 (3.6%), originating from the increased and normal mortality flocks, respectively. Various other serogroups were confirmed for both groups, ranging from 0.5% to 2.2% (Table 1). The isolation frequency of O78 and O111 strains in flocks with increased mortality was significantly higher compared with the flocks with normal mortality (*p* < 0.05).

The serogroups classified based on the geographic distribution, type of birds and farm as well asper organ of origin are reported in Table 2, Table 3, Table 4 and Table 5.

Table 3 reveals a differentiation in the serogroup prevalence according to age (*p* < 0.05). The O78 serogroup was predominant in layer chicks/pullet strains whereas the O2 serogroup was detected only in the laying flocks. Table 4 reveals the serogroup variation among the different farms/regions.

### 3.2. Interaction with Infectious Agents

The association of MG, MS, IBV and ILT infections with *E. coli* mortality is presented in Table 6, Table 7, Table 8 and Table 9 while raw data are included in Supplementary files (Spreadsheets S1: MG, MS, IBV, ILT).

#### 3.2.1. *Mycoplasma gallisepticum* and *Mycoplasma synoviae*

*Mycoplasma* serological monitoring revealed a high level of exposure to *Mycoplasma gallisepticum* and *Mycoplasma synoviae*. Regarding the *Mycoplasma gallisepticum* infection level, 78.94% of the flocks with normal *E. coli* mortality were positive against *Mycoplasma gallisepticum* whereas 73.81% of the flocks with increased *E. coli* mortality were positive.

Most of the positive flocks, 42 out of 46, appeared to have all samples positive (10/10) whereas 2 flocks had 9/10 positive, 1 flock had 6/10 positive and 1 flock had 3/10 positive.

Concerning the *Mycoplasma synoviae* infection, the level of infection was even higher. A total of 94% of the flocks with normal mortality were positive whereas the percentage of flocks with increased mortality was 92.85%. Concerning the MS-positive flocks, 56 out of 57 flocks had 10/10 positive serum samples whereas1 flock was seroconverted from 0 positive samples to 2/10.

#### 3.2.2. Infectious Bronchitis

The characterization of the flocks for the IBV infection status is shown in the Table 8. The term ‘suspect for infection’ was used because no definitive diagnosis could be performed.

Concerning the flocks tested, three flocks showed a remarkable increase of the mean titer in combination with respiratory symptoms and abnormal shell eggs whereas three other unvaccinated flocks showed very high antibody titers. Those high titers could indicate an IBV infection according to the Zoetis guidelines.

None of the 14 flocks with normal mortality that were checked for an IBV infection were characterized as suspect for infection. Concerning the 31 flocks with increased mortality, 6 flocks were characterized as suspect for infection (16.21%). Three of these flocks showed a high increase in the antibody titers in the second blood sampling (seroconversion), which could not be explained by the vaccination schedule; it was combined with the clinical symptoms of infectious bronchitis (respiratory and eggshell abnormalities). The other three flocks gave extremely high titers of antibodies and were considered to be suspect for infection according to Zoetis guidelines.

#### 3.2.3. Infectious Laryngotracheitis

The characterization of the flocks for the ILT infection status is shown in Table 9 and the term ‘suspect for infection’ was used in order to indicate a possible infection.

From the ILT infection characterization results, it emerged that in flocks with normal mortality, only 1out of 14 flocks (7.14%) was suspected to have an ILT infection whereas 10 out of 37 flocks were suspected to have an ILT infection (27.02%) among the flocks where *E. coli* was isolated and showed increased mortality. All the flocks that were characterized as suspect for infection had clinical symptoms and tracheal macroscopic lesions suspected for ILT. Eight of these flocks had positive serological results without being vaccinated and three flocks showed a rise in the mean titer at a pair blood sampling.

## 4. Discussion

The presence of untypeable *E. coli* isolates has been reported in previous studies [28,29,30,31,32,33,34,35,36]. Approximately 180 O antigens are used today to characterize the strains in O-serogroups [1]. Therefore, the number of strains that remain untypeable is related to the number of applied monospecific antisera. In the present study, a series of 30 antisera was used in two different labs. The use of a higher number of antisera might reduce the presence of untypeable strains. Nevertheless, in studies where all (181) antisera were applied for *E. coli* characterization, several isolates still remained untyped [37,38,39]. The reason for unsuccessful typing could be the presence of surface antigens such as the K antigen in the bacterial cell of *E. coli* that inhibits the agglutination of the O antigen [26]. Furthermore, it is not possible to serotype rough strains that can autoagglutinate and the presence of new serotypes that have not yet been identified remains a possibility [1].

In our study, *E. coli* of serogroups O78 and O2 showed that these were the most prevalent serogroups identified among the flocks with increased mortality in both pullets and layers, which was in agreement with previous studies [29,32,33,35,36,40,41,42,43,44,45,46,47,48], confirming their predominance in many parts of the world and in both broilers and layers with colisepticemia. Furthermore, a variety of *E. coli* serogroups manifested between increased and normal mortality flocks, which has also been established in different research projects [34,41,42,49]. However, O78, O2 and O1 serogroups are not always the most prevalent serogroups of APEC strains in epidemiological studies. In contrast to our findings, various other serogroups have been reported to be predominant [30,39,50,51].

The presence of O111 *E. coli* strains in birds with colibacillosis has been previously demonstrated. Zanella et al. [52] detected O111 isolates in layers with colibacillosis and polyserositis in Italy. Srinivasan et al. [53] found that the O111, O166 and O64 *E. coli* serogroups were the most common ones in layers with egg peritonitis in India whereas another study in the U.S. also found O111 and O78 strains in layers with peritonitis [54]. Furthermore, Giovanardi et al. [55] managed to detect an *E. coli* strain that was assigned to the O111 serogroup in a turkey suffering from colibacillosis and Khalifa et al. [56] reported the isolation of O111 *E. coli* strains in one-week-old broiler chicks with omphalitis. Finally, Mora et al. [57] revealed the presence of two emerging clonal serogroups of the O111 serogroup, emphasizing the increasing occurrence of this serogroup during recent years in Spain.

Other serogroups that were identified in our study have also been previously reported in studies of poultry suffering with colibacillosis such as O88 [33,46], O8 [46,50], O1 [40,43,44,47,48], O18 [31,43,47,48], O45 [46,48], O103 [31], O5 [30,42], O15 [30,35] and O147 [42]. Several serogroups were exclusively present in the increased mortality flocks (O78, O111, O18, O1 and O103) or within the normal mortality group (O147, O15 and O5) where as several serogroups were detected in both groups of birds (O2, O88, O8 and O45). These findings are in agreement with several research projects where common O-serogroups were isolated both from birds with normal mortality and birds with clinical colibacillosis [34,37,43]. Furthermore, Rodriguez-Siek et al. [31] attempted to characterize and compare APEC and fecal strains, reporting that several isolates from each category were assigned to unshared serogroups; however, common serogroups were detected in both the APEC and fecal strains. To summarize our findings with relevant studies conducted at broiler farms, the high prevalence of the O78 and O2 groups was in accordance with similar references from research projects performed on broiler poultry. The presence of less common serogroups such as O111 [56,57] has also been reported in broilers similar to various other serogroups [32,34,37,42] or untypeable strains [28,32,34].

The pathogenicity of *E. coli* strains is attributed to different virulence factors. There is a huge diversity in the virulence factors of APEC strains. Virulence factors are divided into adhesins, iron acquisition systems, invasions, toxins and protectins [58] and although it seems that there is no specific APEC genotype for all strains, it has been reported that certain virulence factor patterns are more likely to be detected in APEC strains compared with non-pathogenic *E. coli* strains. As a result, many different serogroups might include pathogenic strains [7,40,59] and strains that belong to the same serogroup could differ in pathogenicity [60]. Several serogroups such as O78 and O2 have been found to include strains that more often contain certain virulence patterns andthus have a pathogenic action. This explains why those serogroups are more prevalent in studies investigating APEC serogroups. The presence of an increased number of virulence factors or of certain virulence factor patterns that are responsible for *E. coli* pathogenicity in serogroups O78 and O2 has been reported in various research projects [31,40,43,61,62]. Similar findings have been reported for pathogenic *E. coli* strains that belong to the O111 serogroup. Yaguchi et al. [34] revealed that O111 isolated strains were homogenous regarding their virulence gene pattern and consistent with the important virulence factors of APEC strains. It seems that the virulence factor characterization might contribute to the identification of APEC strains.

Finally, in the present study, we observed a different serogroup occurrence trait of two serogroups (O78, O2) between the two age-dependent groups of birds (rearing and laying period). The O78 serogroup was mainly found in young birds in comparison with the O2 serogroup that was detected only in the laying flocks. On the contrary, Dias da Silveira et al. [30] observed no differences in the presence of *E. coli* serogroups of isolates between day-old broiler chicks and adult broilers with colibacillosis. Similar findings of shared serogroups between different aged groups of birds were reported by Paudel et al. [63].

Our attempt to find an association between *Mycoplasma* and colibacillosis revealed a high prevalence of both *Mycoplasma gallisepticum* and *Mycoplasma synoviae* infections in the layers. However, no statistically significant difference was recorded between *Mycoplasma gallisepticum* infections and increased mortality (*p* = 0.66) due to colibacillosis. Similar findings were observed for *Mycoplasma synoviae* infections (*p* = 0.78).

The data for *mycoplasma* prevalence in poultry vary from country to country. Nevertheless, *Mycoplasma synoviae* infections in layers seem to be high in many countries (Germany: 95%; U.S.: 84%; UK: 78%) [64]. A Dutch survey reported that the surveillance program for *Mycoplasma synoviae* infections in commercial layer flocks revealed 73% positive flocks [65]. A Belgian study in commercial layers with *E. coli* infections revealed a high level of bird flock infections from *Mycoplasma synoviae* whereas none of the flocks were infected by *Mycoplasma gallisepticum* [41].

Our findings were in accordance with several other research projects that revealed no significant interaction between *Mycoplasma gallisepticum/synoviae* infections and colibacillosis outbreaks [41] or revealed that colibacillosis can occur in mycoplasma-free birds [66]. On the other hand, several studies have established the predisposing role of *Mycoplasma* [19,67,68]. *Mycoplasma gallisepticum* or *synoviae* infections may play a role in stimulating colibacillosis mortality. However, bird exposure to *mycoplasmas* is not necessarily followed by a colibacillosis outbreak because in our study, both MG and MS showed a high prevalence in the normal mortality flocks. This trait couldbe explained by the fact that *mycoplasma* can infect poultry and remain in a latent state, waiting for the appropriate combination of infectious agents or environmental factors to cause clinical disease [27]. Furthermore, the role of MS in respiratory infections is not always pronounced.

Concerning the role of IBV and ILT, no statistically significant difference was observed between the flocks with normal and increased *E. coli* mortality. However, it seemed that the flocks that were possibly infected with IBV tended to have a higher risk of mortality related to an *E. coli* infection because the six flocks suspected for an IBV infection had increased *E. coli* mortality. Similar findings were reported for ILT where the occurrence of infection was higher in the increased *E. coli* mortality groups. This could be explained by the pathogenic action of IBV and ILT damaging the respiratory epithelium and thus facilitating the establishment of the *E. coli* infection.

In contrast to our findings, the relationship of IBV and *E. coli* has been reported in various research projects [11,69,70,71] as well as the association between ILT infections and colibacillosis [17,72]. However, our data about IBV and *E. coli* infections were in agreement with [41], who reported no significant relationship between those two agents.

## 5. Conclusions

To the best of our knowledge, this study provides, for the first time, an insight into the prevalent *E. coli* serogroups circulating in commercial layer and layer breeder flocks in Greece. Although several serogroups identified in our study such as O78 and O2 have frequently been reported to include APEC strains, less common serogroups such as O111 were also detected. This finding indicated that many different serogroups could be associated with colibacillosis and further molecular epidemiological studies should be executed to unravel specific APEC characteristics. Furthermore, these data can be used to develop more efficient intervention strategies against colibacillosis such as vaccination schemes because vaccination stimulates a mainly homologous protection against the used vaccine serotype.

Our investigation on the association between *E. coli* mortality and certain infectious agents such as MG, MS, IBV and ILT did not reveal a statistically significant effect of those specific infectious agents on increased mortality related to an *E. coli* infection in layer poultry flocks. However, the triggering role of other infectious agents cannot be excluded. Therefore, further investigations should take place because a better understanding of the various predisposing factors can help in the effective prevention of *E. coli* infections and thus allow antimicrobial use to decrease in poultry production.

## Figures and Tables

**Table 1:** Summary of *E. coli* strain results according to flock mortality trait.

			Flock Mortality Trait *			
	Total		Normal Mortality		Increased Mortality	
O-Serogroup	No. of Strains	% of Total No.	No of Strains	% of Total No.	No of Strains	% of Total No.
O78	16	6.9		0 ^a^	16	9.1 ^b^
O2	15	6.5	4	7.2 ^b^	11	6.2 ^b,c^
O111	15	6.5		0 ^a^	15	8.5 ^b^
O88	6	2.5	2	3.6 ^a^	4	2.2 ^a,c,d^
O8	4	1.7	1	1.8 ^a^	3	1.7 ^a,d^
O45	3	1.2	1	1.8 ^a^	2	1.1 ^a,d^
O147	2	0.8	2	3.6 ^a^		0 ^d^
O103	1	0.4		0 ^a^	1	0.5 ^a,d^
O18	1	0.4		0 ^a^	1	0.5 ^a,d^
O15	1	0.4	1	1.8 ^a^		0 ^a,d^
O5	1	0.4	1	1.8 ^a^		0 ^a,d^
O1	1	0.4		0 ^a^	1	0.5 ^a,d^
Serogrouped	66	28.6	12	21.9	54	30.7
Untypeable	165	71.4	43	78.1	122	69.3
Total No. Of Strains	231	100	55	100	176	100

Different superscript letters (a, b, c, d) within a column and different O-serogroups indicate a statistical significance (*p* < 0.05). * A mortality percentage of 0.1% per week was regarded as normal.

**Table 2:** Geographical distribution of serogrouped *E. coli* strains.

	Serogroup													
Regions	O78	O2	O111	O88	O8	O45	O147	O1	O18	O5	O15	O103	% Grouped Strains	No. of Samples
Attica		10	4	3	1		2			1			31.82 ^a^	66
Sterea Ellada	15	1	10		3	2		1	1		1		29.31 ^a^	116
Central and Western Macedonia		2	1	2									41.67 ^a^	12
Thrace and Eastern Macedonia	1			1									8.33 ^b^	24
Thessaly		1				1							66.67 ^a^	3
Crete													0.00 ^a^	1
Peloponnesus													0.00 ^a^	2
Epirus												1	100.00 ^a^	1
South Aegean Sea		1											20.00 ^a^	5
Northern Aegean Sea													0.00 ^a^	1

Different superscript letters (a, b) within a column and different areas indicate a statistical significance (*p* < 0.05).

**Table 3:** Distribution of *E. coli* strains according to type of birds.

	Type of Birds					
	Rearing Farms: Pullets		Commercial Layers		Layer Breeders	
Number of Samples	65		149		17	
Untypeable Samples	39		110		16	
Serogroups	No.	%	No.	%	No.	%
O78	15	23.1 ^a^	1	0.7 ^b^		0.0 ^b^
O2		0.0 ^b^	15	10.1 ^c^		0.0 ^b,c^
O111	4	6.2 ^c^	11	7.4 ^c^		0.0 ^b,c^
O88	1	1.5 ^b^^,c^	4	2.7 ^b^^,c^	1	5.9 ^b,c^
O8	3	4.6 ^b^^,c^	1	0.7 ^b^^,c^		0 ^b^^,c^
O45		0.0 ^b^^,c^	3	2.0 ^b^^,c^		0 ^b^^,c^
O147		0.0 ^b^^,c^	2	1.3 ^b^^,c^		0.0 ^b^^,c^
O103	1	1.5 ^b^^,c^		0.0 ^b^^,c^		0.0 ^b^^,c^
O18		0.0 ^b^^,c^	1	0.7 ^b^^,c^		
O15		0.0 ^b^^,c^	1	0.7 ^b^^,c^		
O5	1	1.5 ^b^^,c^		0.0 ^b^^,c^		
O1	1	1.5 ^b^^,c^		0.0 ^b^^,c^		

Different superscript letters (a, b, c) within a column and different O-serogroups indicate a statistical significance (*p* < 0.05).

**Table 4:** Distribution of *E. coli* strains between farms with typeable serogroups.

Farm	Type of Birds	Region	O-Serogroup (*n*)	Farm	Type of Birds	Region	O-Serogroup
No3	LB	A	O88	No24	CL	SE	O2
No4	RFM	SE	O78 (7), O8 (2), O1 (1)	No27	CL	CWM	O2
No5	RFM	SE	O78 (8), O111 (2)	No32	CL	CWM	O88
No6	RFM	TEM	O88	No34	CL	CWM	O2
No7	CL	TEM	O78	No38	CL	A	O2
No8	CL	SE	O45	No39	CL	T	O2, O45
No10	CL	SE	O45	No42	CL	SE	O15
No11	CL	A	O111 (2)	No43	CL	A	O2
No12	CL	A	O2 (5)	No44	CL	A	O147 (2)
No13	CL	SE	O111 (4)	No46	CL	A	O88
No15	CL	SAS	O2	No49	RFM	SE	O111 (2)
No18	CL	A	O2 (2)	No50	RFM	A	O5, O8
No19	RFM	E	O103	No51	RFM	A	O111 (2)
No21	CL	CWM	O111, O88	No53	CL	A	O2
No22	CL	SE	O111 (2)	No59	CL	SE	O18, O8
No23	CL	A	O88				

Type of birds: rearing farm pullets (RFM), commercial layers (CL), layer breeders (LB). Region: Attica (A), Sterea Ellada SE), Central and Western Macedonia (CWM), Thrace and Eastern Macedonia (TEM), Thessaly (T), Crete (C), Peloponnesus (P), Epirus (E), South Aegean Sea (SAS), Northern Aegean Sea (NAS).

**Table 5:** Distribution of *E. coli* serogroups in relation to organ origin(number of strains).

O-Serogroup	Pericardium/Liver	Peritoneum	Air Sacks/Trachea	Yolk Sack
O78	14	0	2	0
O2	7	8	0	0
O111	12	1	2	0
O88	4	2	0	0
O8	0	1	2	1
O45	0	3	0	0
O147	0	2	0	0
O103	1	0	0	0
O18	0	1	0	0
O15	0	1	0	0
O5	0	0	0	1
O1	0	0	1	0

**Table 6:** Status of MG infection among sampled flocks (MG: *Mycoplasma gallisepticum)*.

Flock Colibacillosis Mortality	MG Infection		
	No	Yes	Total
Normal	4	15	19
Increased	11	31	42
Total	15	46	61

**Table 7:** Status of MS infection among sampled flocks (MS: *Mycoplasma synoviae*).

Flock Colibacillosis Mortality	MS Infection		
	No	Yes	Total
Normal	1	18	19
Increased	3	39	42
Total	4	57	61

**Table 8:** Status of IBV infection among sampled flocks (IBV: infectious bronchitis virus).

Flock Colibacillosis Mortality	Suspect for IBV Infection		
	No	Yes	Total
Normal	14	0	14
Increased	31	6	37
Total	45	6	51

**Table 9:** Status of ILT infection among sampled flocks (ILT: infectious laryngotracheitis).

Flock Colibacillosis Mortality	Suspect for ILT Infection		
	No	Yes	Total
Normal	13	1	14
Increased	27	10	37
Total	40	11	51

## Data Availability

The data presented in this study are available on request from the corresponding author.

## Supplementary Materials

The following supporting information can be downloaded at: https://www.mdpi.com/article/10.3390/vetsci9040152/s1, Spreadsheet S1: MG, MS, IBV, ILT.
